# Does watching a movie improve empathy? A cluster randomized controlled trial

**Published:** 2019-11-28

**Authors:** Azin Ahmadzadeh, Mehdi Nasr Esfahani, Masoud Ahmadzad-Asl, Mohammadreza Shalbafan, Seyed Vahid Shariat

**Affiliations:** 1 School of Medicine, Iran University of Medical Sciences, Tehran, Iran; 2 Mental Health Research Center, School of Behavioral Sciences and Mental Health (Tehran Institute of Psychiatry), Iran University of Medical Sciences, Tehran, Iran

## Abstract

**Background:**

We studied if watching a movie about the patient physician encounter alone or in combination with a communication skills training workshop could improve empathy score of medical students.

**Methods:**

One hundred and thirty three medical students participated in one of the following four groups of the study. Group A: a three hour workshop (42 students); group B: watching the movie “The Doctor” (23 students); group C: watching the movie “The Doctor”, then, participating in a three hour workshop the next day (22 students); group D: control group with no intervention (46 students). Participants completed Jefferson Scale of Empathy (JSE), Student Version to assess empathy score before and after the intervention, and one month later. A linear mixed effect model analyzed the effect of intervention across groups considering the effects of other significant variables.

**Results:**

All of the three interventions had an immediate improving effect on empathy scores compared to control group. However, the improvement effect remained significant only in groups A (p=.015) and C (p=.001) one month later.

**Conclusions:**

Watching selected movies has a significant but transient effect on empathy of students. Combining two methods of watching the movie and communication skills workshop, seems to add the beneficial effects.

## Abstract (fr)

**Contexte:**

Nous avons étudié si le fait de regarder un film sur une rencontre patient-médecin seule ou en association avec un atelier de formation sur les compétences en communication pouvait améliorer le score d’empathie des étudiants en médecine.

**Méthodes:**

Cent trente-trois étudiants en médecine ont participé à un des quatre groupes suivants de l’étude. Groupe A : un atelier de trois heures (42 étudiants); groupe B : regarder le film « Le Docteur» (23 étudiants); groupe C : regarder le film « Le médecin » et ensuite participer à un atelier de trois heures le jour suivant (22 étudiants); groupe D : groupe témoin sans intervention (46 étudiants). Les participants ont rempli l’échelle d’empathie de Jefferson (JSE), version étudiante, pour évaluer le score d’empathie avant et après l’intervention, ainsi qu’un mois plus tard. Un modèle d’effet mixte linéaire a analysé l’effet de l’intervention parmi les groupes en tenant compte des effets d’autres variables significatives.

**Résultats:**

Les trois interventions ont un effet d’amélioration immédiate sur les scores d’empathie comparativement au groupe témoin. Toutefois, un mois plus tard, l’effet d’amélioration n’était resté significatif que dans les groupes A (p = 0,015) et C (p = 0,001).

**Conclusions:**

Regarder des films sélectionnés a eu un effet significatif, mais transitoire sur l’empathie des étudiants. Combiner les deux méthodes, regarder le film et suivre un atelier sur les compétences en communication, semble ajouter les effets bénéfiques.

## Introduction

“The surgeon’s work is to cut…”, said Dr. McKee to his students in an influential scene of the movie “The Doctor”, trying to show how a surgeon should deal with emotions in an encounter with a patient.^1^ Maybe many physicians still think as Dr. McKee and seek to keep emotions away for the sake of objectivity. However, this kind of attitude toward physician- patient relationship might adversely affect the quality of patient’s care and undermine the formation of an empathic therapeutic relationship.

There has been a great deal of inconsistency in definition of empathy in the literature and important efforts have been done to review how empathy is defined in medical education research.^2^ Hojat defines empathy as “a predominantly cognitive (as opposed to affective or emotional) attribute that involves *understanding* (as opposed to feeling) of the patient’s experiences, concerns, and perspectives, and a capability to *communicate* this understanding and an intention to help”.^3^ Because of the beneficial effects of empathy in various outcomes of physician-patient encounter,^4^ many studies have attempted to improve empathy in health professionals and students. This is especially important because many studies have reported that empathy score of medical students decreases with increasing years of education.^5^ However, there are other studies that cast a doubt on the aforementioned decline of empathy^6^ and even another study promise an improvement in some aspects of students’ empathy with increasing years of education.^7^

Researchers have empirically validated at least ten methods for their positive effect on empathy. These methods include “improving interpersonal skills, audio- or video-taping of encounters with patients, exposure to role models, role playing (aging game), shadowing a patient (patient navigator), hospitalization experiences, studying literature and the arts, improving narrative skills, theatrical performances, and the Balint method”.^8^ Generally speaking, these enhancement methods try to either improve interpersonal skills of the participants or involve the trainees in an experience of disease and results in better understanding of the patients’ problems, or both of them.

In other words, the methods used to improve empathy work on the ability of the participants to “understand” patients’ experiences and emotions and their ability to “communicate” this understanding; the two main concepts that are included in the abovementioned definition of empathy. Role playing, shadowing a patient, hospitalization, theatrical performance, studying literature and the art, and improving narrative skills mainly improve understanding of the participant from the real situations including pain and difficulties that patients experience and help the participant to view the issue from the patient’s perspective. However, interpersonal skills workshops, audio or videotaping of interviews with patients, and Balint method might be more effective in improving communication ability and developing the necessary skills for better rapport. We are aware that this categorization might be an oversimplification and some of the aforementioned methods might affect both abilities simultaneously. However, we choose to label the methods in this way to emphasize the main concept underscored in each of them.

One of the weaknesses of these empathy enhancement methods is limited sustainability of positive changes: the finding of improved ability to empathize declines with time.^9^ Reinforcing an educational method with another one can help to increase the beneficial effects or durability of the positive changes.^10^^,^^11^ Watching films or movies has been successfully used both as a method to improve empathy^12^^,^^13^ and a method combined with another educational method to improve sustainability of the increased empathy.^10^ However, to our knowledge, no randomized controlled trial has yet been published on the effects of watching a movie on empathy and the possible effects of combining it with another augmenting method of empathy.

Therefore, we designed the study to see if watching a movie about the patient physician encounter (The Doctor, 1990)^1^ alone or in combination with a communication skills training workshop could improve the empathy score of medical students. We hypothesized that the combination of the two methods might result in a greater improvement in empathy scores immediately after intervention and a smaller decline in empathy one month later.

## Methods

### Trial design and setting

Medical students who were taking clinical rotations in Rasoul-E-Akram Hospital during January 2016 to February 2017 comprised the study population. Rasoul-E-Akram Hospital is a big hospital complex in Tehran and one of the two main clinical training centers for medical sciences in Iran University of Medical Sciences (IUMS). Medical students take many of their rotations in this hospital.

### Participants

We included all of the wards of the hospital with a total number of 174 medical students in the study. These students were taking their clinical training period from year 4 to 7 of medical training. This clinical period is divided to an initial 2.5 year of externship period and a final 1.5 year of internship period. We used cluster random assignment method to allocate the wards to one of the four arms of the study. Ward administrative staff did not allow 41 students to leave their wards to take part in the study. Therefore, 133 students began the study in one of the following four groups.

**Figure 1:**
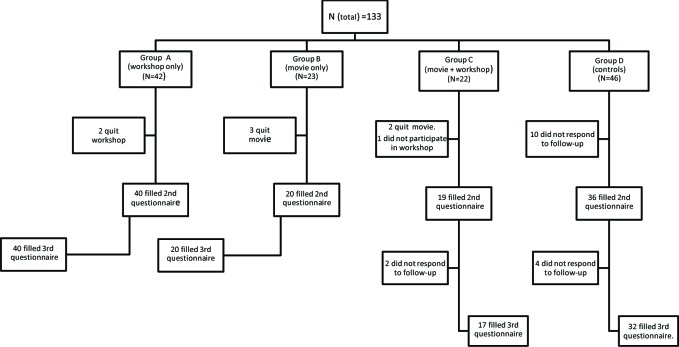
Flow diagram of the trial showing 133 participants allocating to study arms and their progress through difference stages of the trial

- Forty two students in group A: A three hour workshop on communication skills training
- Twenty three students in group B: Watching the movie “The Doctor”
- Twenty two students in group C: Watching the movie “The Doctor”, then, participating in a three hour workshop of communication skills training the next day
- Forty six students in group D: Control group with no specified intervention (Fig. 1)

It is important to note that the number of students taking rotations in different wards of the hospital was not equal. Furthermore, unexpectedly, a number of students were not allowed to participate in the study. Therefore, the number of participants were not equal in the four arms of the study. The number of students that completed the first phase of the study was 115 (18 students did not fill the second questionnaire) (more details in Fig. 1). Six students did not complete the one-month later follow up questionnaire and 109 students completed the study (82% retention rate).

### Interventions

Communication Skills Training workshop: An associate professor of psychiatry with several years of experience in teaching communications skills (MNE) taught the workshop. The workshop began with a question about personal experience of the participants as a patient with doctors or health services, and about how this experience might have affected them. Then, he introduced the concept of patient-physician relationship, therapeutic relationship and empathy and its importance and discussed them with students. Finally, we showed a short role-play film depicting two different types of patient-physician interactions and discussed about the positive and negative points in each of the role- plays.

“The Doctor”: It is an American movie directed by Randa Haines with a running time of 122 minutes (released in 1991). The story is about an arrogant cardiac surgeon (Dr Jack MacKee) who is diagnosed with laryngeal cancer and this new experience of illness provides him with fresh insight into patient- physician relationship. We showed the movie in the amphitheater of the hospital using a video-projector, in original language (English) with Persian subtitle. It is important to note that we performed the interventions (workshop and movie) of the different arms of the study independently and in different days but in the same amphitheater and with similar conditions.

### Outcome measurement

Our main outcome measure was empathy of the students. We assessed the empathy score of the participants with Jefferson Scale of Empathy (JSE), Student Version at three time points. First, after allocation to the groups and before any intervention. Second, immediately after the intervention, or for the control group two to three hours after the first assessment. Third, one month after assignment to the groups.

JSE is a validated self-report scale that is specifically designed to assess empathy in health professionals and their related students.^3^^,^^14^^,^^15^ It includes 20 items that are scored from 1 (strongly disagree) to 7 (strongly agree) in a Likert-type scale. The scale has been previously translated and validated in Persian.^16^^,^^17^ Additionally, we added a number of demographic questions, including gender, age, marital status, and educational level (externship or internship) to the beginning of JSE.

### Ethical issues

This study was approved by the Ethical Committee of Iran University of Medical Sciences (Code:IR.IUMS.REC 1395.9311286001). We were committed to the Declaration of Helsinki and ethical rules of our country throughout the study. We informed the participants that we would consider the data confidential and questionnaires are anonymous. Participation was voluntary and the participants were able to withdraw without any consequences at any step of the study. Participants also received two gifts: one mug after first post-test and one flash-memory after 1-month follow-up test, at the end of the study. This study has also been registered as a clinical trial in Iranian Registry of Clinical Trials (Code: IRCT 2016082629534N1).

### Statistical analysis

We used SPSS (Statistical Package for the Social Sciences) - version 16 for data analysis. We used Chi- square test to compare qualitative variables and analysis of variance (ANOVA) to compare quantitative variables between the groups. Due to the difference of the outcome variable at baseline, we calculated the differences of empathy scores observed in each group from baseline to second observation (intervention effect) and from second to third observation (stability of intervention effect) in order to observe the possible changes in empathy scores.

Considering the advantages of mixed effect models over traditional ANOVA models,^18^^,^^19^ we decided to perform a linear mixed effect model analysis instead of a classical repeated measure ANOVA. We used repeated measure ANOVA only to have a general demonstration of the changes observed in each group. To measure the independent effect of the study intervention and potential covariates/factors on change in empathy score, we performed a linear mixed model analysis. We set the empathy score as the dependent variable and group, gender, educational level, passing psychiatric rotation, and time, as well as interactions of group*time, and group*gender as fixed effects and time as random effect and baseline empathy score as covariate in the primary model. To form the final model we excluded the variables that did not show a significant effect in the primary model (passing psychiatry rotation, educational level and group*gender interaction), and calculated parameter estimates of fixed effects and estimates of covariance parameters according to the final model. We calculated Cohen’s d as an index of effect size of the intervention.^20^ P value of <.05 was considered as statistically significant.

## Results

The mean age of the participants was 24.7 (SD=1.5). Forty three (37.4%) participants were male and seventy two (62.6%) were female. Seventy-eight (67.8%) participants had taken the psychiatry rotation before the beginning of the study. Forty two (36.5%) participants were passing their externship training and 73 (63.5%) their internship training. There was a statistically significant baseline difference in age, educational level and passing psychiatry rotation between the four groups (Table 1).

**Table 1:** Baseline characteristics of 133 participants in the four arms of the trial

		Total	Group A	Group B	Group C	Group D	Statistical sig.
Age (years)	Mean(±SD)	24.7(±1.5)	24.7(±1.4)	24.0(±1.4)	24.3(±1.2)	25.4(±1.4)	≤ 0.001
Gender N (%)	Male Female	43(37.4%) 72(62.6%)	13(32.5%) 27(67.5%)	5 (25.0%) 15(75.0%)	8 (42.1%) 11(57.9%)	17(47.2%) 19(52.8%)	0.336
Passing psychiatry rotation N (%)	Yes No	78(67.8%) 37(32.2%)	25(62.5%) 15(37.5%)	10(50.0%) 10(50.0%)	11(57.9%) 8 (42.1%)	32(88.9%) 4 (11.1%)	0.009
Educational Level N (%)	Externship Internship	42(36.5%) 73(63.5%)	17(42.5%) 23(57.5%)	10(50.0%) 10(50.0%)	12(63.2%) 7 (36.8%)	3 (8.3%) 33(91.7%)	≤ 0.001
Marital status N (%)	Single Married	91(79.1%) 24(20.9%)	28(70.0%) 12(30.0%)	19(95.0%) 1 (5.0%)	15(78.9%) 4 (21.1%)	29(80.6%) 7 (19.4%)	0.164

### Empathy score

**Figure 2:**
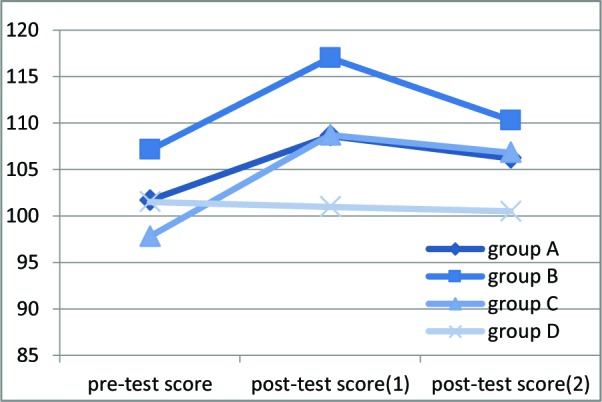
Changes observed in JSE score at three time points before and after the intervention and one month later on 133 medical students.

The mean empathy score (based on JSE) for all of the participants before interventions were 101.9 (SD=12.2) and there was no significant baseline empathy score differences between the four groups, but immediately after interventions, the mean increased to 107.7 (SD=12.3), and this intervention effect was different between groups (Figure 2). All of the active intervention groups showed an increase in JSE score, but group D (control group) did not show a significant change. The increase was more prominent in groups B and C. However, at 1-month follow up, JSE score decreased to 105.4 (SD=10.9). This decline was again observed in all three active intervention groups and was more pronounced in group B (film only). Decline of JSE score was not present in the control, group D (Table 2) (Fig.2).

**Table 2:** Mean score of JSE before (baseline score) and after intervention (post-test 1) and one month later (post-test 2) and its statistical significance based on repeated measure ANOVA in 133 participants of the four arms of the trial

	Baseline score	Post-test 1	Post-test 2	Statistical sig.
	Mean (SD)	Mean (SD)	Mean (SD)	
Group A	101.7 (10.8)	108.6 (10.9)	106.2 (10.3)	P=0.004
Group B	107.1 (9.4)	117 (11.2)	110.3 (9.8)	P=0.104
Group C	97.8 (14.6)	108.7 (8.7)	106.8 (7.8)	P=0.006
Group D	101.5 (13.1)	101 (12.6)	100.5 (12.4)	P=0.8
Total	101.9 (12.2)	107.7 (12.30)	105.4 (10.9)	

We used linear mixed effect model analysis to the measure independent effect of study intervention and potential covariates/factors. The primary model included empathy score as dependent variable and group, gender, educational level, passing psychiatric rotation, and time, as well as interactions of group*time, and group*gender as fixed effects and time as random effect and baseline empathy score as covariate in the primary model. Passing psychiatry rotation, educational level and group*gender interaction did not show a significant effect and were excluded from the final model as suggested by Seltman (chapter 15, p. 369).^19^

In the final model, the following variables showed a significant effect: group (p<.001), time (p<.001), gender (p=.02), baseline empathy score (p<.001) and group*time interaction (p<.001). It shows that each of these variables independently are related with empathy score. However, to understand the difference between the groups and across time, we need to look at parameter estimates of fixed effects (Table 3). In this table, group D, third assessment, has been considered as the index group for comparison in the model and their value have been set to zero.

**Table 3:** Parameter estimates of fixed effects of the variables and interactions in the mixed effect model analysis on a sample of 133 medical students

Parameter	Estimate	t	Sig.
Intercept	22.3	6.1	<.001
Group A	4.8	3.1	**.002**
Group B	4.6	2.4	**.016**
Group C	8	4	**<.001**
Group D	0^a^	.	.
Pretest	.5	.4	.71
Post-test 1	.1	.1	.93
Post-test 2	0^a^	.	.
Group A * Pretest	-5.1	-2.5	**.015**
Group A * Post-test 1	2.3	1.1	.27
Group A * Post-test 2	0^a^	.	.
Group B * Pretest	-3.7	-1.5	.13
Group B * Post-test 1	6.6	2.6	**.009**
Group B * Post-test 2	0^a^	.	.
Group C * Pretest	-9	-3.5	**.001**
Group C * Post-test 1	2.3	.9	.37
Group C * Post-test 2	0^a^	.	.
Group D * Pretest	0^a^	.	.
Group D * Post-test 1	0^a^	.	.
Group D * Post-test 2	0^a^	.	.
Female	1.9	2.3	**.022**
Male	0^a^	.	.
Pretest score of empathy	.8	22.3	**<.001**

a This parameter is set to zero because it is redundant.

Mean score of empathy was different between group D and all of the other groups regardless of time of assessment (first four rows of Table 3). Time variable is shown not to be associated with empathy score (next three rows of table 3). The main finding of this table is the significant interaction of group and time on empathy score. It means that empathy score has changed differently during time in different groups. Interaction of time and groups A and C shows that empathy score in post-test 2 is significantly higher than pretest (group A: p=0.015, group C: p=0.001), but not significantly different from post-test 1 (group A: p=0.27, group C: p=0.37). It means that in Group A and C, empathy has significantly changed from first to second assessment, but there is no significant change from the second to the third assessment. In other words, empathy improved in “workshop only” group (group A) and film and workshop group (group C) and did not significantly decline one month later. However, in group B the score of empathy in post-test 2 is significantly lower than post-test 1 (p=0.009), but not different from pretest score (p=0.13). It means that empathy has increased in group B from the first to second assessment and has again declined one month later. The final rows of Table 3 show the significant independent effect of gender and pretest score of empathy on dependent variable.

Finally, estimates of covariance parameters was not significant in the model (Wald Z=1.9, p=.052). Therefore, the model did not support the presence of a random effect for the variation of empathy score in each participant across different assessments. In other words, the findings does not confirm the presence of an unmeasured explanatory variable that might change the performance of each participant in a seemingly random way in different assessments.

### Effect size

We measured Cohen’s d index for groups A and C that showed a significant improvement of empathy after one month (table 4). We calculated effect size using the first and third assessments [Cohen’s d = (M3- M1)/pooled SD]. Group A had a small to moderate effect size (.43) but group C showed a medium to large effect size (.77).

**Table 4:** Intervention effect and stability of intervention and the calculated effect size of the intervention in 133 participants of the four arms of the trial

	Intervention effect	Stability of intervention	Net effect	Cohen’s d
	Mean (SD)	Mean (SD)		
GroupA	6.9 (8.2)	-2.4 (8.7)	4.5	.43
Group B	9.9 (8.6)	-6.7 (8.7)	3.2	-
Group C	10.9 (12)	-2.6 (4.9)	8.3	.77
Group D	-.4 (6.8)	-.2 (10)	-.2	-
Total	5.8 (9.6)	-2.6 (8.8)	3.2	

Intervention effect: change in JSE score observed between baseline and second assessment immediately after intervention; Stability of intervention: change of JSE score from second to third assessment showing the decline after one month of follow up.

## Discussion

The study shows that all of the three interventions (communication skills workshop, watching the movie, and workshop plus watching the movie) have an immediate positive effect on empathy scores of medical students compared to control group. However, watching the movie seemed to increase the immediate effect and participating in the workshop tended to decrease the decline of the score during the follow up and so appeared to improve the sustainability of the effect of the intervention.

In this study we tried to evaluate the effect of augmenting the most widely studied method of empathy improvement i.e., communication skills training with watching a movie. We expected that watching the movie would increase the motivation of the participant to learn from the workshop and in this way would increase the beneficial effect of education on empathy. However, the study findings did not support this hypothesis, at least for its short-term effects that we assessed immediately after the intervention. Combination of the two methods (workshop and movie) showed a larger effect size compared to the “workshop only” group, but the difference was not statistically significant. Interestingly, the two groups who watched the movie had a steeper immediate increase in their empathy scores. Therefore, it seems that short-term effects of watching the movie might even be more powerful than participating in the workshop; but the two effects are not additive at this time point.

Why this happens might be due to different mechanisms by which the two methods affect empathy. Movies engage participants emotionally with the story and make them identify with characters. This emotional involvement and identification with movie characters might be the underlying mechanism through which empathy is enhanced. Because it prepares an opportunity for the participants to share the experience of the movie character and get familiar with his problems to some extent. This involvement helps the participants to improve their “understanding” of the patient’s experience. Gladstein put forward this concept as “[v]iewers lose themselves in the film to the extent that they are not conscious of their surroundings. These ideas closely parallel Lipps’s beliefs about empathy”.^21^

On the other hand, a communication skills workshop defines the related concepts and helps the participants learn the definition and implications of empathy and its positive effects on treatment outcomes and how it can be used in encounters with patients. Several studies have shown the effectiveness of these workshops in improving empathy of the participants.^22^^,^^23^ These workshops generally employ a variety of techniques, including lecture, audio or visual presentation of educational material, and role playing to teach basic communication skills (recognition patient’s emotions and communicating them in a constructive way) to the intended audience.^8^^,^^22^ Therefore, workshop provides more explicit and structured learning about the physician-patient relationship than the movie. It seems that the “movie only” group have experienced a kind of arousal and increased attention to the subject area of the movie, which is patient-physician relationship and so, have an increased score in JSE. However, the lack of formal teaching in the “movie only” group did not allow the students to benefit from their increased attention and learn a new skill. This assumption might explain why the “workshop only” group had a better sustainability and a smaller decline in empathy one month later compared to the “movie only” group.

Finally, when we augment structured learning provided by the workshop with watching the movie, we may profit from the merits of both methods, i.e., emotional involvement that results in a steeper increase in empathy, and structured learning that results in a slower decline of empathy. Therefore, the net effect of combining workshop and movie would be a larger effect on empathy and a larger mean difference compared to workshop. Combination of instructional and experiential methods has also been used by Bayne to improve empathy in medical students.^24^ Interestingly, the effect size of this study has been reported to be larger than the other studies reviewed in a systematic review (mean effect size of 15 articles=.23).^25^ Combination of strategies has also been used as an augmentation method to boost and increase the sustainability of previous education. For example, Hojat et al. showed that augmenting a first intervention to improve empathy (watching and discussing video clips about patient encounters) with an upcoming lecture on empathy would increase the sustainability of the intervention effect on empathy score of medical students.^10^

These findings suggest that combining various educational methods could have beneficial effects and remove some of the shortcomings of the current known methods. Herein, workshops have an exceptional potential for combining different methods. As mentioned above, many different presentation methods, other than lecture, including audio or visual presentation of educational material, role-playing, and theatrical performance have been integrated into communication skills workshops to enrich them and augment their positive effects on empathy.

Our study has some strengths and limitations. Design of the study with three intervention and one control group made it possible to examine reliably the effect of watching the movie in isolation or in combination with communication skills training workshop. Furthermore, using a well validated scale and powerful statistical methods are other strengths of the study.

However, we only conducted the study in one center, which limits the generalizability of the findings. Furthermore, study groups were different in the level of education and empathy score at baseline. Level of education was not independently associated with empathy score; therefore, we do not think that the difference in level of education would have caused a noticeable problem. Moreover, we included the baseline scores of JSE as a covariate in the model and controlled for its possible effect. The other limitation of our study is the fact that we did not randomly allocate each participant to the study groups. Instead, we randomly assigned the study clusters. This is an acceptable alternative method when individual allocation of the participants is not possible. Finally, the movie was shown in English with Persian subtitles; presentation of a Persian translated version of the movie might have been more effective.

### Conclusion

Showing movies depicting patient-physician encounters and related issues to medical students seems to have beneficial effects on learning of empathy, when combined with communication skills workshops. We suggest that medical schools consider using this method; because it is not only a socializing and enjoyable activity, but also an inexpensive method that can be easily administered. Future studies can make use of other creative ways to increase the effect size or sustainability of the changes or develop practical programs that can be integrated into curriculum of medical education.^26^
